# Social prescribing and behaviour change: proposal of a new behaviour change technique concerning the ‘connection’ step

**DOI:** 10.1080/21642850.2021.2019584

**Published:** 2022-01-05

**Authors:** Kathryn B. Cunningham, Rayna H. Rogowsky, Sharon A. Carstairs, Frank Sullivan, Gozde Ozakinci

**Affiliations:** a Population and Behavioural Sciences, School of Medicine, University of St Andrews, St Andrews, UK; b School of Health Sciences, University of Dundee, Dundee, UK

[S]ocial prescribing … creates the opportunity for real and lasting behaviour change. (Health Education England, 2016)

Social prescribing, also known as community referral, is gaining international recognition as a health and social care initiative with benefits for individuals (e.g. improving mental wellbeing), healthcare services (e.g. decreasing inpatient admissions), and societies (e.g. creating more inclusive communities) (Drinkwater, Wildman, & Moffatt, 2019; Global Social Prescribing Alliance, 2021). Further, it is being advocated as an advantageous tool to help facilitate recovery from the COVID-19 pandemic (Royal College of Psychiatrists, 2021). Social prescribing involves a health or social care professional connecting an individual with an appropriate community-based opportunity to improve that individual’s health (physical and/or mental) and wellbeing (physical, mental and/or social), for example, a local jogging group to increase physical activity levels, or a hobby club to reduce feelings of loneliness. The connection can be made via a direct route (health or social care professional connecting person to community-based opportunity) or an indirect route (health or social care professional connecting person to social prescribing professional – usually referred to as ‘link worker’ or ‘community connector’ – and social prescribing professional connecting person to community-based opportunity). Different methods of connection can be utilised: signposting, prescription or referral in a direct route, and a combination of these methods in an indirect route (Cunningham, Rogowsky, Carstairs, Sullivan, & Ozakinci, 2021). Social prescribing can therefore be conceptualised as a ‘system’ (Husk, Elston, Gradinger, Callaghan, & Asthana, 2019, p. 7) comprising two parts: (1) the community-based opportunities for health and wellbeing improvement; (2) the processes of connection from health or social care to those community-based opportunities (Cunningham et al., 2021; Husk et al., 2019).

Social prescribing is recognised as a useful approach for facilitating behaviour change (Health Education England, 2016). Behaviour change interventions employing social prescribing firstly enhance an individual’s motivation to change, then connect the individual with an appropriate community-based opportunity to assist with turning motivation into action. Social prescribing therefore deserves attention in the field of behaviour change. The *connection* step of behaviour change interventions employing social prescribing is not currently included in the widely-used Behaviour Change Technique (BCT) Taxonomy (Michie et al., 2013), nor in additional BCT taxonomies focussing on particular behavioural domains (e.g. the CALO-RE taxonomy for physical activity and health eating behaviours [Michie et al., 2011]). The inclusion of the *connection* step in these BCT taxonomies is warranted. The purpose of these BCT taxonomies is to facilitate development, implementation and evaluation of, and evidence synthesis regarding, behaviour change interventions by providing a standardised terminology to enable precise and clear specification and coding of the active ingredients of such interventions (BCTs) (Michie et al., 2013; Scott et al., 2020). The taxonomies currently contain BCTs enabling specification and coding of active ingredients employed to enhance an individual’s motivation to change, e.g. *Information about health consequences*, and active ingredients to assist with turning motivation into action, e.g. *Instruction on how to perform a behaviour*. However they do not contain a BCT enabling specification and coding of the *connection* step of behaviour change interventions employing social prescribing. This absence hinders comprehensive specification and coding of the active ingredients of such interventions. The taxonomies pre-date the recent upsurge of interest in social prescribing, thus this gap is unsurprising. Literature regarding the taxonomies acknowledges the need for them to be updated as new evidence emerges and new BCTs are identified: “*We anticipate that further refinement and development of BCT Taxonomy v1 will occur as a result of its use and feedback from primary researchers, systematic reviewers and practitioners*” (Michie et al., 2013, p. 93); “*a descriptive taxonomy of BCTs is not written in stone. Additional iterations are needed to optimise reliability, comprehensiveness, theoretical coherence and relevance*” (Michie et al., 2011, p. 1482).

We therefore believe it timely – given the increasing momentum of social prescribing and its potential to facilitate recovery from the COVID-19 pandemic – to propose a new BCT enabling specification and coding of the *connection* step of behaviour change interventions employing social prescribing. We present this new BCT in the format of the BCT Taxonomy version 1 (Michie et al., 2013) in Table 1.

**Table 1.** The label, definition and examples of the new BCT.

Label	Definition	Examples
*Connecting with community-based opportunity*	*Connect the person with a community-based opportunity to facilitate performance of the desired behaviour via a direct or indirect route and using signposting, prescription, referral or a combination of these methods*	Formally refer the person to a jogging group in the local community Provide the person with an information leaflet about a weight loss support group and advise that they attend

We envisage that this BCT will allow comprehensive specification and coding of the active ingredients of behaviour change interventions employing social prescribing. In doing so it will facilitate development, implementation and evaluation of, and evidence synthesis regarding, such interventions, thereby promoting progress in the fields of behaviour change and social prescribing. We recommend that this BCT be considered for inclusion in the BCT taxonomies as they are updated – we plan to engage with the team(s) responsible for the taxonomies regarding this, as suggested in published advice (Michie et al., 2013).
